# Burden and Risk Factors of Urinary Schistosomiasis and Bacteriuria Coinfections Among Schoolchildren in an Endemic District in the Ashanti Region, Ghana

**DOI:** 10.1155/japr/4632076

**Published:** 2026-10-12

**Authors:** Patrick Frimpong, Denis Dekugmen Yar, Nana Yaa Awua-Boateng, Ebenezer Assoah, Kofi Addo Agyapong, Kwaku Addai, Christian Amponsah

**Affiliations:** ^1^ Department of Public Health Education Asante Mampong Campus University of Skills Training and Entrepreneurial Development (AAMUSTED) Asante Ghana; ^2^ Department of Biological Sciences Education Asante Mampong Campus University of Skills Training and Entrepreneurial Development (AAMUSTED) Asante Ghana

**Keywords:** bacterial infections, coinfection, Ghana, schoolchildren, urinary schistosomiasis

## Abstract

This study assessed the prevalence and risk factors for urinary schistosomiasis coinfection with bacteriuria among schoolchildren in an endemic District of the Ashanti Region of Ghana. A cross‐sectional study design was employed to assess the prevalence and coinfection of urinary schistosomiasis with bacteriuria among 312 participants. Urine samples were collected from each participant and examined microscopically for *Schistosoma haematobium* eggs and also cultured for bacteriuria. The overall prevalence of urinary schistosomiasis among the participants was 27.2%. Children aged 12–13 years had the highest rate (33.8%, *p* = 0.04), and males had a higher rate (37.5%, *p* = 0.01) than females (16.4%). Users of streams/rivers and boreholes as water sources had schistosomiasis rates of 40.1% and 31.7%, respectively. Prevalence rates of 42.9%, 32.5%, and 28.2% were recorded among children who swam, fished, or used stream water for domestic activities, respectively. The majority of schoolchildren (51.6%) had bacteriuria, with *Escherichia coli* (27.9%) and *Staphylococcus aureus* (18.6%) being the predominant isolates. The prevalence of coinfection of urinary schistosomiasis with bacteriuria among schoolchildren was 17%, with *E. coli* (28.3%) and *S. aureus* (18.9%) the most common causes. Swimming in rivers/streams and being male increase the odds of schistosomiasis infection by 2.00 (95% CI = 1.50–2.70, *p* = 0.01) and 3.20 (95*%*CI = 2.80–3.80, *p* = 0.04), respectively. Water‐contact activities, such as fishing (OR = 1.35, 95% CI = 0.82–2.23, *p* = 0.03) and domestic activities (OR = 1.12, 95% CI = 0.86–1.86, *p* = 0.04), significantly increased the risk of bacteriuria. These findings suggest that these infections were linked to environmental exposure and inadequate hygiene. It is recommended that integrated control strategies that address both schistosomiasis and bacterial coinfections, including regular praziquantel administration, improved sanitation, snail vector control, and school‐based hygiene education, be implemented to curb the transmission of the dual burden in endemic communities.

## 1. Introduction

Urinary schistosomiasis, caused by *Schistosoma haematobium*, and classified as a neglected tropical disease, remains a significant public health concern in sub‐Saharan Africa, particularly among rural populations [1]. Globally, over 650 million people are at risk of schistosomiasis, with at least 253.7 million people infected and requiring preventive treatment [2], of which at least 93.9% live in Africa [2]. In Ghana, the disease is prevalent in regions with poor sanitation and frequent exposure to contaminated water [3].

Early symptoms of urinary schistosomiasis include blood in urine (hematuria) and painful urination (dysuria) [4]. The sequelae of chronic urinary schistosomiasis infection can lead to anemia, poor growth, reduced school attendance, kidney damage, bladder fibrosis, and even bladder cancer [4, 5]. In girls, genital lesions increase the risk of reproductive health problems and heightened susceptibility to HIV [6].

Although praziquantel remains the mainstay of treatment and is often distributed through school‐based mass drug administration programs, reinfection rates remain high in many communities with unsafe water and poor sanitation [1]. Beyond chemotherapy, improving sanitation, ensuring access to safe water, and intensifying hygiene education are essential for sustainable control [7]. Snail control and behavioral change interventions also play important roles in disease prevention and control. However, these measures are rarely employed in rural communities such as the Sekyere South District [1].

Understanding the drivers of transmission is therefore essential for targeting control efforts effectively. The transmission of *S. haematobium* is influenced by environmental, behavioral, and sociodemographic factors. Limited knowledge of transmission and poor hygiene practices further increase their vulnerability. School‐aged children are at higher risk due to frequent water‐contact activities such as swimming, fetching water, and bathing in rivers, streams, or ponds [3, 7]. Other determinants include family size, source of drinking water, parental education, and toilet facilities [7]. In many endemic communities, lack of health education contributes to low awareness and high reinfection rates [1]. Although national control efforts have reduced schistosomiasis prevalence in Ghana from 21% to below 7% over the past 15 years, hotspots remain, particularly in rural Ashanti and northern Ghana [1, 7].

Another growing concern is the co‐occurrence of urinary schistosomiasis and bacteriuria among school‐aged children. Studies in Ghana and across Africa suggest that children infected with *S. haematobium* are more likely to develop urinary tract infections (UTIs) due to altered immunity and damage to the urinary tract epithelium [5, 8]. For instance, coinfections with bacteria such as *Escherichia coli*, *Salmonella*, and *Staphylococcus* have been reported in endemic areas, worsening morbidity and complicating treatment outcomes [4, 8]. However, limited research exists in Ghana on the epidemiology of urinary schistosomiasis and coinfections with bacteriuria, particularly in rural districts.

Addressing this knowledge gap, it is critical to provide empirical data on the prevalence, coinfection rates, and disease transmission dynamics to inform interventions targeting both parasitic and bacterial infections among vulnerable school‐aged populations. This study seeks to provide up‐to‐date data to complement nationwide surveillance and policy formulation, thereby achieving outcomes aligned with the World Health Organization roadmap to eliminate schistosomiasis by 2030 [9]. Hence, this study is aimed at determining the prevalence and coinfection rates of urinary schistosomiasis with bacteriuria, as well as the risk factors for these infections.

## 2. Materials and Methods

### 2.1. Study Design and Area

#### 2.1.1. Study Design

This study employed a cross‐sectional design to assess the prevalence and risk factors for the co‐occurrence of urinary schistosomiasis and bacteriuria among school children. The study was conducted from January to July 2025 in the Sekyere South District of the Ashanti Region, Ghana (Figure 1).

**Figure 1:**
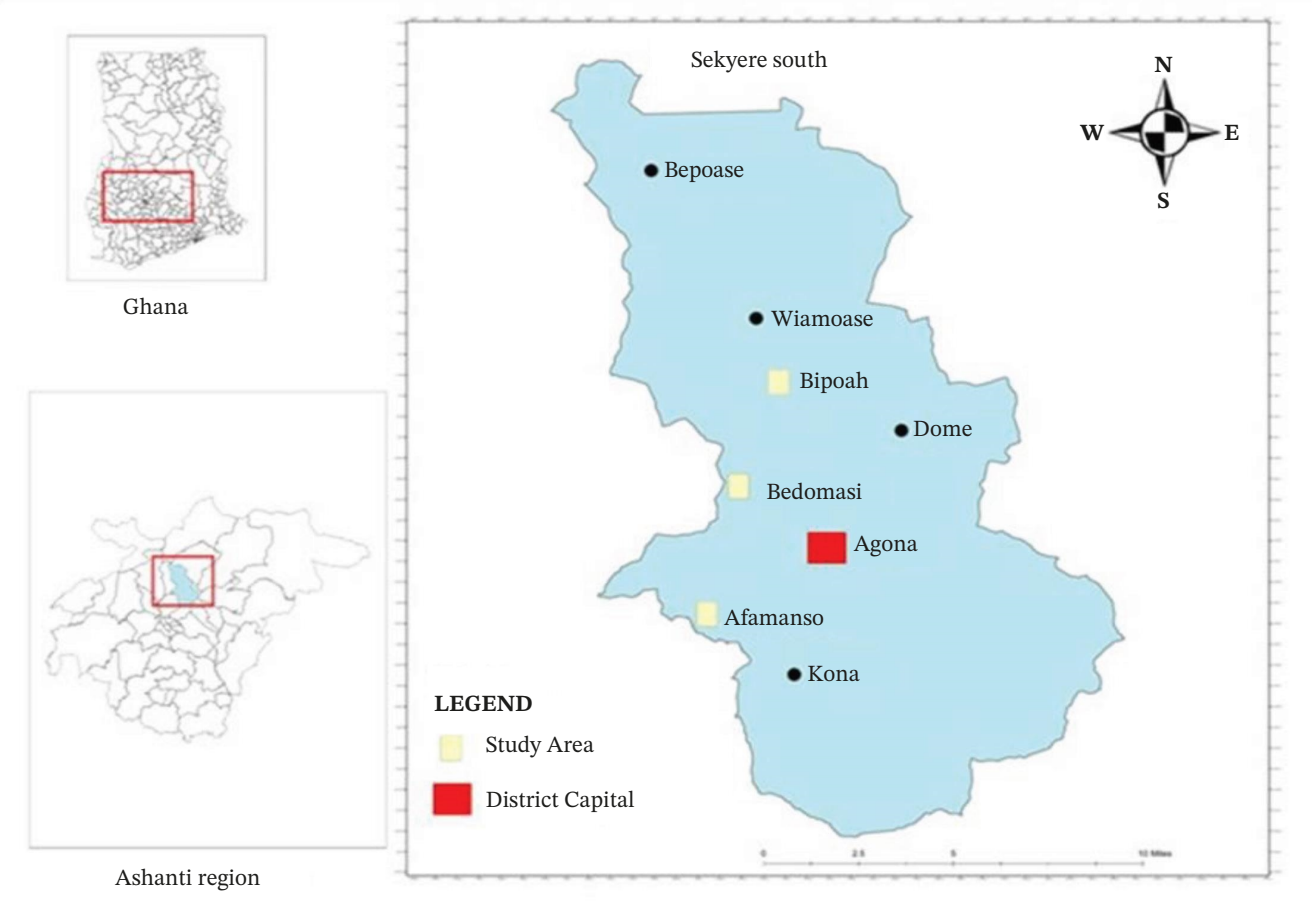
Map of Sekyere South District showing study area (Arc GIS Version 7.1).

#### 2.1.2. Study Area

The district was established in 1988 and has a population of 120,076, representing 3.3% of the regional population (2021 Census). The district′s population density is 293.4 people per square kilometer, and its annual population growth is 2.3%. Urinary schistosomiasis is endemic in the district. Historical data from [10] showed infection rates of 40%–60% among schoolchildren in lakeside and riverine communities, whereas [11] showed a high prevalence in areas and the rural Ashanti region. The disease imposes a considerable health burden, particularly among rural and periurban populations, such as those in the study district.

Sekyere South District is situated between latitudes 6.85°N and 7.35°N and longitudes 1.05°W and 1.30°W in the southern forest zone of Ghana. The district borders Sekyere Central to the north, Afigya Kwabre to the south, and Bosomtwe District to the west. The total land area of Sekyere South is approximately 1226 square kilometers, comprising lowland forest vegetation and hilly terrains. The district has an elevation ranging from 213 to 290 m above sea level, which supports freshwater bodies and human settlements near water.

Sekyere South experiences a bimodal rainfall pattern, with major rains from March to July and minor rains from September to November each year. The average annual rainfall ranges from 1250 to 1500 mm, resulting in stagnant pools that are ideal for snail breeding and the transmission of schistosomiasis (SSDA‐2023; GSS‐2021). The average temperature ranges from 24°C to 29°C, with relative humidity varying between 65% and 95% throughout the year. The wet season favors the growth of aquatic vegetation, which supports the snail intermediate hosts (*Bulinus* species) of *S. haematobium* parasites (SSDA‐2023; GSS‐2021). Most communities rely heavily on unprotected water sources such as rivers, streams, and ponds for bathing, drinking, washing, and recreational activities. Water bodies such as the Offin River, Oyon River, and several stagnant pools serve as major transmission sites in the district. The lack of household latrines and improper waste disposal contribute to fecal and urinary contamination of surface waters in these communities (SSDA‐2023; GSS‐2021). These environmental and behavioral factors make Sekyere South a suitable location for studying the transmission of urinary schistosomiasis and associated risk factors.

### 2.2. Ethical Considerations

Ethical approval was obtained from the Committee on Human Research, Publications and Ethics (CHRPE), KNUST (Approval No: CHRPE/AP/010/2025). The Sekyere South Education Directorate and the headteachers of the selected schools granted official permission. Written informed consent was obtained from parents or guardians. Verbal assent was also obtained from all eligible children before any procedure began. All personal information was kept confidential. Questionnaires and samples were labeled with ID codes rather than names to maintain anonymity. Participation was voluntary. Children could withdraw from the study at any point without any consequences, such as discrimination or loss of treatment.

### 2.3. Study Population, Inclusion, and Exclusion Criteria

The study included schoolchildren aged 6–15 years, ensuring age homogeneity and alignment with standard schistosomiasis surveillance protocols. Kindergarten 2 through junior high school students at six selected public basic schools within the district were selected for the study. This age group was targeted because of their high risk of schistosomiasis infection from frequent water contact activities such as swimming, fishing, or wading through open water bodies. Participation required parental consent and child assent. Children with overt and previously diagnosed schistosomiasis, who are on treatment with praziquantel within the past 6 months, as well as those on antibiotic treatment and those with serious medical conditions, were excluded. Participants who did not get parental consent were also excluded.

### 2.4. Sample Size Determination

Based on this population, a sample of 350 was estimated using [12, 13] sample size determination at 95% confidence level, a margin of error of 0.05, and a 5% nonresponse rate [1, 7]. However, samples from 312 participants were appropriate for examination and analysis.

### 2.5. Sampling Procedure

The study district was stratified into eight zones, of which six covered three communities and were purposively selected due to the presence of rivers, streams, and ponds. One school situated near water bodies was also purposively selected from each of the selected zones, using the district′s school register. The number of participants per school was determined based on the school′s population size. Participants were stratified by class and age, and the number in each stratum was determined by the total enrollment across Kindergarten 2 through junior high school 3. Class registers served as sampling frames, and participants were selected using random number tables without replacement. Attendance lists were updated on the day of the survey.

### 2.6. Data Collection Procedures

Data on participants′ demographic characteristics and risk factors were collected using a structured questionnaire. A laboratory‐designed form was used to collect urine samples and record laboratory results. Data and urine samples were collected from the study participants on the school premises by trained research assistants, who administered questionnaires face‐to‐face. The research assistants were laboratory technicians, field technicians, and school teachers. They were trained on the general study and, more specifically, the sampling and data collection procedures for 2 days. Responses were recorded on precoded forms labeled with unique participant identifiers. Data collected included sociodemographic characteristics, water‐contact activities, access to sanitary facilities at home, a history of hematuria, and knowledge of schistosomiasis transmission. The questionnaire was prepared in English and translated into Twi. The translation was validated by two local teachers and pretested at a nonparticipating school.

### 2.7. Urine Sampling

Each participant provided a midstream urine sample between 10:00 a.m. and 2:00 p.m., after a few minutes of physical exercise. This is the peak period of egg excretion in urine. Sterile, wide‐mouthed urine containers (PlastLab, Batch No: PL‐041024GH) were distributed to each participant. The research team instructed participants on how to collect urine directly into the containers without contamination. Each container had a preprinted barcode and child identification number. Urine samples were placed in Coleman cooler boxes with ice packs and transported within 4 h to the parasitology laboratory for examination and analysis.

### 2.8. Laboratory Procedures

#### 2.8.1. Urine Analysis and Examinations

In the laboratory, each sample was examined macroscopically for color, bloodstains, and turbidity under fluorescent light. This was immediately followed by streaking each urine sample on three different culture media and incubating for 24 h. The remaining urine samples were subsequently processed for *S. haematobium* ova.

#### 2.8.2. Urine Microscopic Examinations

Each urine sample was thoroughly mixed with a sterile pipette and transferred into a 15 mL conical centrifuge tube (Thermo Fisher, Batch No. TF‐C15‐0525). Samples were centrifuged using Hettich Universal 320 centrifuge (Batch No: HET‐U320‐2023) at 3000 revolutions per minute (rpm) for exactly 5 min. The supernatant was carefully poured off. The remaining sediment was then transferred to a Sail Brand microscope slide (Batch No: SB‐MS0425) using Eppendorf pipettes.

A clean coverslip (Lab Coat, Batch No: LC‐CS2025) was gently placed on the sample. The slide was examined using a Leica DM500 light microscope. The microscope (Leica DM500, Serial No. DM5‐L‐03024) was calibrated daily using calibration slides before each use. Magnification levels of 10x and 40x were used. *S. haematobium* eggs were identified by their terminal spine and oval shape. The number of eggs per 10 mL of urine was recorded. Infection intensity was categorized as light (1–49 eggs/10 mL) or heavy (≥ 50 eggs/10 mL), in accordance with the WHO 2023 classification guidelines [6]. Each sample was examined independently by two experienced microscopists. A third expert technician reviewed any disagreements in the two readings. Slides were stored in slide storage boxes (SterilTech, Batch No: STBX‐25‐0425) for verification and future quality checks. These procedures were designed to ensure compliance with quality standards. A urine sample was negative after examination of at least 100 fields, with no *S. haematobium* ova observed. The observations and counts were recorded accordingly.

### 2.9. Isolation and Identification of Bacteria From Urine

#### 2.9.1. Media Preparation

Sterile agar (cysteine lactose deficiency agar—CLED agar), blood agar, and MacConkey agar were prepared to support bacterial growth as previously reported [14, 15]. These media were prepared, autoclaved, and then poured into sterile Petri dishes according to the manufacturer′s guidelines [16].

#### 2.9.2. Culture, Isolation, and Identification

Bacteria from urine samples were isolated by inoculating cystine lactose deficient (CLED) agar plates and incubating them for 24 h at 37°C. Colonies were examined for morphological characteristics, and pure cultures were obtained by subculturing isolated colonies onto nutrient agar. Preliminary identification of bacterial isolates was conducted by subculturing on differential and selective media, including mannitol salt agar (MSA), MacConkey agar, and eosin methylene blue (EMB) agar, followed by biochemical identification [17, 18].

Bacterial isolates were identified based on morphological characteristics (colony morphology and Gram staining), cultural characteristics (growth on selective and differential media) and biochemical characteristics (oxidase, catalase, and coagulase tests), following standard microbiological protocols to ensure accurate identification for epidemiological analysis and treatment planning [14, 18–20]. The biochemical test results were compared with established bacterial profiles in databases to validate the isolates′ identities [21]. All children with urinary schistosomiasis, bacteriuria, or both were referred to the Ministry of Health and Ghana Health Services health facility for treatment by health professionals.

### 2.10. Data Management and Entry

Data from questionnaires and laboratory results were entered into Microsoft Excel 2016. Two separate clerks performed double entry to minimize errors. The lead researcher conducted daily data audits. Discrepant entries were identified and corrected by cross‐checking the original forms and laboratory records. Data were entered into Microsoft Excel (Version 2016), cleaned, and exported to R (Version 4.2.2) for statistical analysis. All variables used in the analysis were categorical. Some variables, such as schistosomiasis and bacteriuria test results and other risk factors, were binary (e.g., positive/negative). In contrast, other variables, such as age group and education level, had more than two levels. All missing variables were managed using a multiple imputation procedure.

### 2.11. Quality Control

Microscopes were cleaned and recalibrated before each session. Centrifuges were tested for speed using a digital RPM meter (Velocity Check VC100, Batch No: VC‐0525). All pipettes and laboratory tools were autoclaved daily using an Autoclave Classic 2100 (Batch No: AC‐2100/0425) at 121°C for 15 min. All laboratory personnel wore disposable gloves, surgical masks, and lab coats (3 M Healthcare, Batch No: 3 M‐LC0425). Gloves were changed between each sample.

### 2.12. Statistical Analysis

Descriptive statistics were used to determine the frequencies and percentages of schistosomiasis and bacteriuria, as well as other socio‐demographic and behavioral characteristics of respondents. The coinfection rate was calculated by dividing the number of individuals positive for both infections (schistosomiasis and bacteriuria) by the total study population (312), and expressed as a percentage:

Coinfection rate=number positive for both infectionstotal study population×100%.

The chi‐square test of independence and binary logistic regression were used to assess the association between infection and other variables. Prior to conducting logistic regression, model fit was evaluated using the Hosmer–Lemeshow goodness‐of‐fit test (*p* > 0.05, indicating a good fit). Additionally, the Nagelkerke *R* ^2^ statistic and the model′s classification accuracy were examined to assess the model′s explanatory power and predictive ability [22].

For the multivariate logistic regression analysis, all variables with *p* values < 0.2 in the univariate analysis were considered for inclusion. Variables with more than two levels were included as categorical factors using dummy coding in RStudio. Variables of known epidemiological importance were also retained in the final model regardless of statistical significance. A *p* value < 0.05 and a 95% confidence interval were used to determine statistical significance.

## 3. Results

### 3.1. Demographic Characteristics of Study Participants

In Table 1, of the 312 participants enrolled in the study, 23.7% were aged 12–13 years, the majority (51.3%) were male, 36.9% were in the upper primary level (Primary 4–6). It is worth noting that 40.1% of the study participants relied on streams or rivers as their primary water source, 18.9% practiced open defecation, and 41.0% resided in Bipoah (Table 1).

**Table 1:** Demographic characteristics among schoolchildren.

Characteristics	Frequency (*n*)	Percentage (%)
Age range (years)		
6–7	55	17.6
8–9	64	20.5
10–11	70	22.4
12–13	74	23.7
14–15	49	15.7
Sex		
Male	160	51.3
Female	152	48.7
Education level		
Kindergarten 2	22	7.1
Primary 1–3	95	30.4
Primary 4–6	115	36.9
Junior high 1–3	80	25.6
Water sources		
Stream/river	125	40.1
Borehole	99	31.7
Piped water	88	28.2
Sanitation facility		
Flushed toilet	98	31.4
Pit latrine	155	49.7
Open defecation	59	18.9
Study community		
Bipoah	128	41.0
Bedomasi	123	39.4
Afamanso	61	19.6

### 3.2. Prevalence of Schistosomiasis Among Schoolchildren

In Table 2, the overall prevalence of schistosomiasis among pupils was 27.2%, with rates of 33.8%, 30.6%, and 18.2% recorded among pupils in the 12–13, 14–15, and 6–7‐year age ranges, respectively (*p* value = 0.04). Boys had the highest prevalence, 37.5% (*p* value = 0.01), and 31.1% among pupils in junior high. The high rates and risks of schistosomiasis infection among school pupils were associated with swimming (42.9%) and fishing (32.5%) in rivers/streams.

**Table 2:** Prevalence of urinary schistosomiasis among pupils.

Characteristics	Infections (*n*)	Percentage (%)	*X* ^2^	*p*
Overall prevalence	85	27.2		N/A
Age range (years)				0.04
6–7	10	18.2	4.69	
8–9	15	23.4		
10–11	20	28.6		
12–13	25	33.8		
14–15	15	30.6		
Sex				0.01
Male	60	37.5	6.17	
Female	25	16.4		
Education level				0.12
Kindergarten 2	5	22.7	5.45	
Primary 1–3	25	26.3		
Primary 4–6	30	26.1		
Junior high 1–3	25	31.3		
Sanitation facility				0.05
Flushed toilet	25	25.5	5.56	
Pit latrine	50	32.3		
Open defecation	10	16.9		
Water contact activities				0.01
Swimming	30	42.9	6.49	
Fishing	25	32.5		
Domestic activities	20	28.2		
Washing cars/bicycles	10	19.6		
Wading in water	5	16.1		

Figure 2 shows urinary schistosomiasis infection rates among the study communities: Bipoah (31.25%), Bedomasi (24.39%), and Afamanso (24.59%).

**Figure 2:**
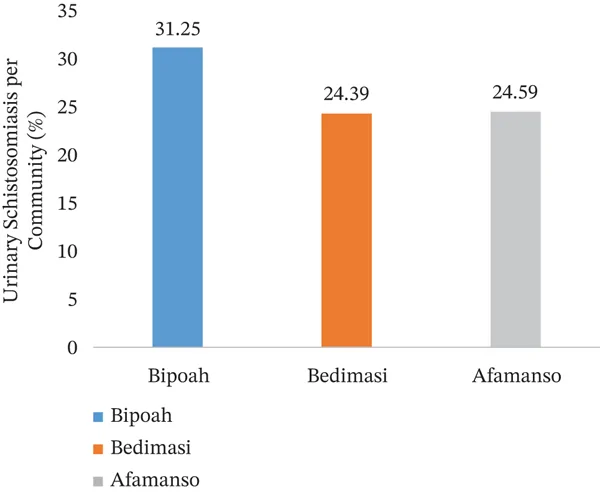
Prevalence of urinary schistosomiasis per community.

Table 3 shows that pupils aged 6–7 years had an increased risk of contracting urinary schistosomiasis (OR = 1.42, 95% CI = 1.08–1.89, *p* = 0.02). Swimming in rivers/streams and being male increase the odds of schistosomiasis infection by 2.00 (95% CI = 1.50–2.70, *p* = 0.01) and 3.20 (95% CI = 2.80–3.80, *p* = 0.04), respectively.

**Table 3:** Association of demographic characteristics and risk factors with urinary schistosomiasis.

Characteristic	Positive infections	Odds ratio (OR)	95% confidence interval (CI)	*p*
Age range (years)				
6–7	10	1.42	1.08–1.89	**0.02**
8–9	15	0.76	0.57–0.99	0.54
10–11	20	1.05	0.88–1.25	0.91
12–13	25	1.58	1.20–2.07	0.3
14–15	15	Reference		
Sex				
Male	60	3.2	2.80–3.80	**0.04**
Female	25	Reference		
Sanitation facility				
Flushed toilet	25	0.75	0.62–0.95	0.29
Pit latrine	50	1.4	0.90–2.16	0.13
Open defecation	10	Reference		
Water contact activities				
Swimming	30	2.0	1.50–2.70	**0.01**
Fishing	25	1.6	1.09–2.30	0.12
Domestic activities	20	1.2	1.07–1.33	0.5
Washing cars/bicycles	10	0.8	0.68–1.20	0.54
Wading in water	5	Reference		

*Note:* Bolded values indicate statistically significant associations (*p* < 0.05).

### 3.3. Bacterial Isolates Among Schoolchildren

Table 4 presents the distribution of the seven bacterial genera isolated from the schoolchildren studied. *E. coli* was the predominant isolate (27.9%), followed by *Staphylococcus aureus* (*S. aureus*) (18.6%), *Klebsiella* species (15.5%), *Enterococcus* species (12.4%), *Pseudomonas* species (11.2%), and *Streptococcus* species (9.9%), whereas *Proteus* species was the least (4.3%).

**Table 4:** Bacterial isolates among schoolchildren.

Bacterial isolate	Frequency	Infection rate (%)
*E. coli*	45	27.9
*Enterococcus* spp.	20	12.4
*Klebsiella*	25	15.5
*Proteus* spp.	7	4.3
*Pseudomonas* spp.	18	11.2
*S. aureus*	30	18.6
*Streptococcus* spp.	16	9.9

### 3.4. Prevalence of Bacterial Infections Among Schoolchildren

Table 5 shows that the overall prevalence of bacterial infections among school pupils was 51.6%, with rates varying by demographic characteristics. The highest bacterial infection rates were recorded among individuals aged 10–11 years (22.4%) and 12–13 years (23.6%). Rates of 50.9% and 49.1% were observed among males and females, respectively. Pupils in primary 4–6 and 1–3 recorded infection rates of 37.9% and 31.1%, respectively. Prevalence rates of 40.4% and 31.1% were observed among pupils using stream/river water and borehole water, respectively, whereas 24.8%, 26.1%, and 49.1% were observed among those who used flushed toilets, practiced open defecation, and used pit latrines, respectively. Bacteriuria rates of 22.4%, 26.1%, and 23.0% were recorded among fishers, swimmers, and domestic water users, respectively.

**Table 5:** Prevalence of bacterial infections among schoolchildren by demographic.

Characteristics	Infected (*n*)	Percentage (%)	*X* ^2^	*p*
Overall	161	51.6		N/A
Age (years)				
6–7	28	17.4	45.62	0.40
8–9	33	20.5		
10–11	36	22.4		
12–13	38	23.6		
14–15	26	16.1		
Gender				
Male	82	50.9	53.21	0.82
Female	79	49.1		
Education				< 0.01
Kindergarten 2	12	7.5	4.34	
Primary 1–3	50	31.1		
Primary 4–6	61	37.9		
Junior high 1–3	38	23.6		
Water source				
Stream/river	65	40.4	13.53	0.06
Borehole	50	31.1		
Piped water	46	28.6		
Sanitation facility				< 0.01
Flushed toilet	40	24.8	6.75	
Pit latrine	79	49.1		
Open defecation	42	26.1		
Water contact activities				< 0.01
Swimming	36	22.4	7.35	
Fishing	40	24.8		
Domestic activities	37	23 0.0		
Washing cars/bicycle	28	17.4		
Crossing	14	8.7		
Others	6	3.7		

In Table 6, children aged 8–9 (OR = 1.54, 95% CI = 1.21–1.99, *p* = 0.04), 10–11 (OR = 1.68, 95% CI = 1.32–2.52, *p* = 0.02), and 12–13 (OR = 1.75, 95% CI = 1.16–2.63, *p* = 0.01) were significantly associated with bacterial infections. School pupils in Primary 4–6 had a higher risk of infection (OR = 1.60, 95% CI = 1.15–2.50, *p* = 0.03) relative to those in junior high school. Water‐contact activities, such as fishing (OR = 1.35, 95% CI = 0.82–2.23, *p* = 0.03) and domestic activities (OR = 1.12, 95% CI = 0.86–1.86, *p* = 0.04), were significantly associated with bacterial infections.

**Table 6:** Association of demographic characteristics and risk factors with bacterial infections.

Characteristics	Adjusted odds ratio (AOR)	95% confidence interval (CI)	*p*
Age (years)			
6–7	1.32	0.85–2.06	0.22
8–9	1.54	1.21–1.99	**0.04**
10–11	1.68	1.32–2.52	**0.02**
12–13	1.75	1.16–2.63	**0.01**
14–15	Ref.		
Gender			
Male	1.12	0.78–1.61	0.54
Female	Ref.		
Education level			
Kindergarten 2	0.88	0.75–1.41	0.68
Primary 1–3	1.45	0.95–2.10	0.08
Primary 4–6	1.60	1.15–2.50	**0.03**
Junior high 1–3	Ref.		
Sanitation facility			
Flushed toilet	0.62	0.51–1.04	0.15
Pit latrine	1.05	0.88–1.12	0.83
Open defecation	Ref.		
Water contact activities			
Swimming	1.18	0.71–1.96	**0.05**
Fishing	1.35	0.82–2.23	**0.03**
Domestic activities	1.12	0.68–1.86	**0.04**
Washing cars/bicycles	0.98	0.56–1.71	0.94
Wading in water	1.2	0.62–2.32	0.58
Others	Ref.		

*Note:* Bolded *p*‐values indicate statistical significance at (*p* < 0.05). Ref. = reference category.

Table 7 summarizes the prevalence of different bacterial isolates among pupils in the study area: Bipoah, Bedomasi, and Afamanso. An overall prevalence of *E. coli* recorded in Bipoah was 30.0%. *Enterococcus* spp. and *Proteus* spp. were the most dominant in Bedomasi, accounting for 19.2% and 7.7%, respectively. In Bipoah, *Klebsiella* spp. accounted for 25.0% of infections. Afamanso reported the highest prevalence of *Pseudomonas* spp. (14.3%) and *Streptococcus* spp. (12.2%). Overall, Bipoah had the highest number of bacteriuria cases (60), followed by Bedomasi (52) and Afamanso (49).

**Table 7:** Prevalence of bacterial isolates among pupils′ communities.

Infection	Frequency	Study communities		
Bacteria spp.	(*n*)	Bipoah (*n*/*%*)	Bedomasi (*n*/*%*)	Afamanso (*n*/*%*)
*E. coli*	45	18 (30.0)	12 (23.1)	15 (30.6)
*Enterococcus*	20	5 (8.3)	10 (19.2)	5 (10.2)
*Klebsiella* spp.	25	15 (25.0)	5 (9.6)	5 (10.2)
*Proteus* spp.	7	2 (3.3)	4 (7.7)	1 (2.0)
*Pseudomonas*	18	5 (8.3)	6 (11.5)	7 (14.3)
*S. aureus*	30	10 (16.7)	10 (19.2)	10 (20.4)
*Streptococcus*	16	5 (8.3)	5 (9.6)	6 (12.2)
Total	161	60 (100)	52 (100)	49 (100)

### 3.5. Coinfection of Urinary Schistosomiasis With Bacteriuria

In Table 8, the overall coinfection rate of schistosomiasis and bacteriuria among the study participants was 17.0%. Coinfection varied significantly across age groups (*p* = 0.01), with the highest rates observed among participants aged 10–11 years (22.9%) and 12–13 years (21.6%). In contrast, coinfection rates of 16.9% and 17.1% were recorded in males and females, respectively. Coinfection rates differed significantly by educational level (*p* = 0.049), with the highest rate recorded among participants in junior high school (30.0%). Participants who relied on stream or river water had a higher coinfection rate (20.0%) than those who used borehole water (15.2%) or piped water (14.8%). Participants using pit latrines had a 12.9% coinfection rate, whereas rates observed in water‐contact activities were as follows: domestic activities (5.8%), fishing (4.8%), and swimming (4.8%).

**Table 8:** Coinfection of schistosomiasis with bacteriuria associated with the participants′ demographic characteristics.

Demographic characteristic	Coinfection (*n*)	Rate (%)	
**Overall**	**53**	**17.0**	**—**
**Age (years)**			**0.006**
6–7	10	18.2	
8–9	10	15.6	
10–11	16	22.9	
12–13	16	21.6	
14–15	1	2.0	
**Gender**			0.250
Male	27	16.9	
Female	26	17.1	
**Education level**			**0.049**
Kindergarten 2	2	9.1	
Primary 1–3	10	10.5	
Primary 4–6	17	14.8	
Junior high 1–3	24	30.0	
**Water source**			0.180
Stream/river	25	20.0	
Borehole	15	15.2	
Piped water	13	14.8	
**Sanitation facility**			0.120
Flushed toilet	10	10.2	
Pit latrine	20	12.9	
Open defecation	3	5.1	
**Water contact activities**			0.100
Swimming	15	4.8	
Fishing	15	4.8	
Domestic activities	18	5.8	
Washing cars/bicycle	5	1.6	
Crossing	1	0.3	

*Note:* Coinfection rate (*%*) = (number positive for both infections/total study population) × 100*%*. Coinfection rate (*%*) for specific variable (example male) = (male positive for both infections/total number of males) × 100*%*. Bolded p values indicate statistical significance at p < 0.05. Bolded demographic characteristics (age and education level) were significantly associated with coinfection.

Table 9 shows that *Streptococcus* spp. exhibited the highest bacteriuria coinfection rate with urinary schistosomiasis (43.8%), followed by *E. coli*, *Pseudomonas* spp., and *S. aureus* (33.3% each), and *Klebsiella* spp. (32.0%). Other bacteriuria coinfections observed among participants with urinary schistosomiasis included *Proteus* spp. (28.6%) and *Enterococcus* spp. (25.0%).

**Table 9:** Prevalence of coinfection of urinary schistosomiasis with specific bacteriuria.

Bacterial isolate	Number of infections	Coinfection with urinary schistosomiasis (*n*)	Percentage (%)
*E. coli*	45	15	33.3
*Enterococcus* spp.	20	5	25.0
*Klebsiella*	25	8	32.0
*Proteus* spp.	7	2	28.6
*Pseudomonas* spp.	18	6	33.3
*S. aureus*	30	10	33.3
*Streptococcus* spp.	16	7	43.8
Total	161	53	100

*Note:* Coinfection rate (*%*) for specific bacteria isolate = (number of positive for specific isolate and schistosomiasis/total number for specific bacteria isolate) × 100*%*.

## 4. Discussion

The prevalence of urinary schistosomiasis in this study was 27.2%, with the highest rates of 33.8% and 30.6% recorded among pupils aged 12–13 and 14–15 years, respectively. This suggests that older children might experience repeated daily exposure to water‐contact activities. These activities, such as swimming, washing clothes, and fetching water from contaminated sources, increase the risk of infection [23]. The prevalence rate in this study is higher than the rates reported in Ghana and other African countries [24]. In Egypt, 5.3% were reported [25] and in Sudan, 8.7% [26]. However, the prevalence in this study was lower than the 48.2% reported in Nigeria [27]. These differences may result from variations in environmental conditions, sanitation infrastructure, and control programs. In Egypt, lower rates may be attributed to effective public health programs, improved access to clean water, and robust disease surveillance. In contrast, areas in Ghana and Nigeria may lack these systems, resulting in higher infection rates [28].

Age is a consistent risk factor in many studies [29, 30]; Olerimi et al., 2023). Older children often participate in outdoor chores and water‐related recreational activities, which may explain the higher rates of infection among them. However, this study observed that children aged 6–7 years had an increased risk of urinary schistosomiasis relative to older children. The plausible explanation could be that they had a lower prevalence (total existing cases) but a higher risk of infection (new cases) due to differences in immune function, exposure, or population size.

This study found a significant difference in infection rates between boys and girls. Previous studies in Egypt and Sudan reported higher infection rates among boys than girls, in agreement with our study [26, 31]. In some study areas, boys tend to engage more in activities such as fishing and swimming, which increases their exposure [23]. Contrary to this finding, studies in Yemen and Nigeria [27, 32] reported no sex difference, suggesting that some communities exhibit gender‐neutral exposure patterns.

Schoolchildren in the Bipoah community had the highest schistosomiasis rate (31.25%), possibly linked to the presence of numerous water bodies. In the current study, sites were purposively selected to include communities with more rivers, streams, and other water bodies, which could explain the high rate observed. Our study findings further showed that swimming in rivers/streams increases the odds of schistosomiasis infection by 2.00 (95% CI = 1.50–2.70, *p* = 0.01). It could be gleaned that living near rivers or lakes increases the risk, as proximity often entails daily exposure to contaminated water, particularly in rural communities [29, 33]. These findings highlight the importance of improving water infrastructure and promoting hygiene education. Without these, children remain at risk of infection. The implementation of such interventions can significantly lower infection rates and improve child health outcomes in endemic regions [28, 34].

In this study, 51.6% of schoolchildren had bacteriuria, with *E. coli* (27.9%) and *S. aureus* (18.6%) predominantly implicated [35]. The prevalence in this study is much higher than that reported by [36], who found that approximately 15% of children in Ghana have UTIs. Several risk factors contribute to the transmission and occurrence of bacterial UTIs, particularly in school‐aged children. Behavioral factors, such as poor personal hygiene, improper wiping techniques after using the toilet, and delayed urination, increase the risk of bacterial entry and colonization [37]. Early identification and management of these risk factors are crucial for preventing the occurrence and recurrence of UTIs in this population [38].

Furthermore, social factors like low socioeconomic status, overcrowded living conditions, and limited health education contribute to the increased vulnerability of school‐aged children to bacterial UTIs. The high rates of bacterial infection may be attributed to limited access to toilets and handwashing facilities, thereby increasing the risk of fecal‐oral transmission. Also, the use of open water sources, poor food hygiene, and limited knowledge of disease prevention all contribute to the widespread occurrence of infections. The very high rate of bacteriuria in this population should be interpreted in the context of the study area and population. These are rural communities with limited access to healthcare and social amenities, and several ponds, streams, and rivers that are largely contaminated. The sample collection and processing were prone to contamination and could affect the results outside the culture.

*E. coli* is a common uropathogen and causes painful urination, abdominal pain, and kidney complications when untreated (Kumar [39]). The high prevalence of *E. coli* (27.9%) among schoolchildren raises serious public health concerns, particularly in rural areas with limited access to clean water and sanitation facilities. According to WHO [6], the presence of *E. coli* in urine or drinking water implicates fecal contamination and suggests poor sanitation and hygiene practices. In this study, older schoolchildren and water‐contact activities, including swimming, fishing, and domestic activities, were significantly associated with UTIs. Poor sanitation, contaminated water, and unhygienic practices significantly increase the risk of UTI burden among children [40].

Studies from Ghana, Gabon, and Rwanda have also reported high rates of bacterial infections among schoolchildren, confirming the pattern observed in this study [41, 42]. In Uganda, 32% of schoolchildren were reported to have UTIs caused by *E. coli* and *Klebsiella,* bacteria commonly found in environments with poor hygiene [43]. In Kenya, the prevalence rate was 40%, primarily attributable to *E. coli* [44], underscoring the widespread issue across many African countries. In Tanzania, *Streptococcus pyogenes* was detected in 6.9% of schoolchildren [45]. A study in Zambia also reported that 15% of schoolchildren had Salmonella‐associated gastroenteritis [46]. These pathogens can cause long‐term complications such as chronic kidney disease and malnutrition if not treated early.

The implications of these findings are profound, as infected children may miss school due to illness, experience stunted growth, or suffer long‐term health effects. Girls may be particularly affected due to the increased risk of urinary infections related to menstrual hygiene challenges, even though this was not statistically significant. Therefore, urgent efforts are needed to promote sanitation and to provide access to toilets and clean water. Health education programs targeting schoolchildren and their caregivers could improve their knowledge of infection prevention, thereby reducing infection rates.

In this study, the estimated coinfection rate among pupils was 17%, with higher rates among older children and among those who used rivers and streams. *E. coli*, *S. aureus, Pseudomonas* spp., and *Streptococcus* spp. were the common coinfecting bacteria. This finding indicates that environmental exposure remains a key factor in dual infections [22]. *Streptococcus* spp. showed the highest coinfection rate of 43.8%, suggesting that some bacteria may be more opportunistic in the presence of parasitic infection. Findings from studies in Ethiopia and Zambia reported coinfection rates of 15% and 20%, respectively [47, 48], underscoring the prevalence of dual infections in areas with poor sanitation. Children who engage in activities such as swimming or fishing are more likely to be exposed to contaminated water. These behaviors increase their risk of both schistosomiasis and bacterial infections.

In Tanzania, a 25% coinfection rate of schistosomiasis and bacteriuria was reported among immunosuppressed patients, a higher rate than that observed in the current study [49]. This suggests that schistosomiasis weakens the immune system, thereby creating a favorable environment for bacterial growth and recurrent infections. Other studies in Malawi and Uganda reported coinfection rates of 18% and 22%, which are reasonably similar to the current study′s result, confirming the association between helminth infections and enteric bacteria [29, 50]. Moreover, hematuria in schistosomiasis infections provides a suitable medium for bacterial growth, which could explain the dual infections.

These findings imply a compounded health burden on children who may face more severe symptoms, slower recovery, and higher treatment costs. Hematuria and bacteriuria are intrinsically associated, supporting the view that an integrated treatment approach is necessary [51]. Thus, the relationship between schistosomiasis and bacterial infections may extend beyond shared exposure to include compromised immunity [52]. Targeted health interventions that combine deworming, access to clean water, and treatment for bacterial infections are crucial for protecting child health in endemic areas.

The Sekyere South District presents a distinctive epidemiological niche, characterized by riverine areas where water contact remains a daily necessity for children, particularly for bathing, fetching, and recreation, thereby sustaining the risk of schistosomal transmission. Although Ghana′s national schistosomiasis prevalence has declined substantially over time, pockets of higher transmission persist. A recent impact assessment indicates a residual prevalence of 6.8% at the national level (down from a baseline of 21.1%) following sustained mass drug administration efforts [7], which is far lower than the 27.2% rate reported in this current study. In periurban local settings, urogenital schistosomiasis, with a prevalence of 15.5%, is reported to undermine the reduction at the national level and is a drawback, not guaranteeing elimination [53]. This heterogeneity makes reactive, site‐adapted surveillance and control essential in districts such as Sekyere South.

The prevalence documented in this study casts doubt on the feasibility of achieving the Ghana Health Service′s goal and the WHO target for schistosomiasis elimination by 2030 [54]. The continuous exposure of children to infested waters, combined with inadequate sanitation infrastructure and limited snail control, makes the complete elimination of this disease unlikely. However, with intensified control strategies including regular praziquantel administration, environmental interventions, and improved access to safe water, significant reductions in morbidity remain realistic goals [53].

A significant contribution of this study is the identification of coinfections between urinary schistosomiasis and bacteriuria. A large proportion of bacteriuria cases were coinfected. This finding suggests that the urinary tract damage and hematuria caused by *S. haematobium* may create an environment that facilitates bacterial colonization [1]. The relatively high proportion of coinfections underscores an additional layer of disease burden among affected children, with implications for both clinical management and community‐level control strategies. The novelty of this evidence in the Sekyere South District underscores the importance of broadening schistosomiasis research to include secondary infections.

### 4.1. Study Limitation

Although most parents and their children provided consent/assent to participate in the study, 10.86% were unable to provide an adequate urine sample for analysis. One limitation of this cross‐sectional study was the lack of clarity regarding whether bacterial infections were directly linked to schistosomiasis. Furthermore, no medical records were available from community healthcare facilities, making it challenging to verify past diagnoses or health histories for the children in the study. This study employed only microscopic examination of urine samples for *S. haematobium* ova, using morphological identification. However, this has limited diagnostic sensitivity and specificity, as well as for species identification, with a high tendency to miss the presence of a few ova in samples. The study did not employ more sensitive and specific diagnostic tools, such as molecular techniques, which could have improved outcomes. The study did not include antibiotic susceptibility testing. This was a field‐based study, and during sample collection, the schoolchildren may not have strictly adhered to aseptic techniques, making the collected samples prone to contamination, which could affect the results outside the culture. Hence, the results of this study should be interpreted in the context of the study area and population. Nonetheless, the study findings provide strong evidence of the coexistence of urinary schistosomiasis and bacteriuria among schoolchildren, underscoring their association. The outcomes would contribute to the national efforts to eliminate schistosomiasis in the very near future.

### 4.2. Implications for Control and Treatment

The implications of these findings are far‐reaching. Control programs must adopt an integrated approach that addresses both schistosomiasis and bacterial coinfections. Although praziquantel remains essential, bacterial pathogens such as *E. coli, S. aureus*, and *Klebsiella* require appropriate antibiotic therapy, with antimicrobial resistance closely monitored. Coinfection worsens health outcomes and negatively impacts pupils′ development and cognitive function. Integrated control measures are urgently needed to improve sanitation, access to safe water, and school‐based health education, thereby reducing infection rates and protecting children′s health. Broader interventions, including environmental management, health education, and snail control, are equally crucial in breaking the transmission cycle. Ultimately, these integrated measures offer the best chance of reducing the dual burden of schistosomiasis and bacteriuria in endemic communities [55, 56].

## 5. Conclusion

The prevalence of urinary schistosomiasis among schoolchildren was high, with swimming in rivers/streams and male sex as significant risk factors for the infection. The prevalence of bacteriuria was very pronounced among schoolchildren, with water‐contact activities, such as fishing, and domestic activities significantly increasing the risk. Although a high coinfection rate of urinary schistosomiasis and bacteriuria was observed among schoolchildren, this was not associated with age, gender, education level, water sources, sanitation facilities, or water‐contact activities. Sanitation conditions and hygiene practices, however, play a significant role in the transmission of these infections among school pupils. These findings underscore the need for targeted, community‐specific public health interventions to address the unique, concurrent challenges posed by urinary schistosomiasis and bacteriuria at the study site. Stakeholders should integrate bacteriuria screening into the district′s existing schistosomiasis control programs to combat dual infections. It is recommended that integrated control strategies that address both schistosomiasis and bacterial coinfections, including regular praziquantel administration, improved sanitation, snail vector control, and school‐based hygiene education, be implemented to curb the transmission of the dual burden in endemic communities.

## Author Contributions

D.D.Y. played a pivotal role in conceptualizing and developing the manuscript, provided critical edits, contributed to data analysis and interpretation, and ensured the manuscript′s intellectual rigor and clarity. P.F. was responsible for data collection, data analysis, and manuscript drafting. N.Y.A‐B. reviewed the manuscript and approved it for publication. E.A. contributed to data analysis and interpretation, managed the data, and reviewed and approved the manuscript for publication. K.A. provided critical edits, internal assessment, and critical feedback on the manuscript. K.A. reviewed the manuscript and approved it for publication. C.A. contributed to the data collection and analysis and reviewed the manuscript.

## Funding

No funding was received for this manuscript.

## Disclosure

All authors have thoroughly read and approved the final manuscript for publication.

## Consent

We (the authors) have all read the final manuscript and consented to its publication.

## Conflicts of Interest

The authors declare no conflicts of interest.

## Data Availability

The data that support the findings of this study are available on request from the corresponding author. The data are not publicly available due to privacy or ethical restrictions.
